# Embitterment and Aggression in Psychotherapy Patients

**DOI:** 10.1192/j.eurpsy.2022.1920

**Published:** 2022-09-01

**Authors:** M. Linden

**Affiliations:** Charité University Medicine Berlin, Psychosomatic Medicine And Rehabilitation, Berlin, Germany

**Keywords:** aggression, embitterment, Psychotherapy

## Abstract

**Introduction:**

Embitterment is an emotion which is known to everybody in reaction to injustice, humiliation, and breach of trust. In greater intensity it can cause severe suffering for the affected person and the social envirnment, can result in lasting impairment, and even lead to dysfunctional behavior, including aggression. Embittered patients need therapeutic help and are regularly seen in psychotherapy. The problem is often not properly recognized, because of the multiple accompanying symptoms and accusations against the environment.

**Objectives:**

Goal of the present study was to learn about the prevalence of embitterment in psychotherapy patients

**Methods:**

Outpatients in routine psychotherapy filled in the PTED scale (post-traumatic embitterment disorder selfrating scale), the K-FAF (short assessment of aggression) and the SCL-90 (symptom-checklist-90). Additionally, sociodemographic data were available.

**Results:**

Included were 118 patients, of whom 22% showed a relevant severity of embitterment, 23.7% a relevant score for reactive aggression, and 54.2% a relevant score for irritable aggression. There was a significant correlation between the PTED scale and the aggression scale.

**Conclusions:**

The data show that embitterment and related aggression are frequent phenomena in psychotherapy patients. Therapists should be aware of this emotion and take proper action to diagnose embitterment and aggressive ideation, which are often covered by other complaints. Special treatments are needed, as the aggressive and negativistic features of embitterment complicate the psychotherapy process.

**Disclosure:**

No significant relationships.

